# Current State of Milk, Dairy Products, Meat and Meat Products, Eggs, Fish and Fishery Products Authentication and Chemometrics

**DOI:** 10.3390/foods12234254

**Published:** 2023-11-24

**Authors:** Slim Smaoui, Maria Tarapoulouzi, Sofia Agriopoulou, Teresa D’Amore, Theodoros Varzakas

**Affiliations:** 1 Laboratory of Microbial, Enzymatic Biotechnology, and Biomolecules (LBMEB), Center of Biotechnology of Sfax, University of Sfax-Tunisia, Sfax 3029, Tunisia; 2 Department of Chemistry, Faculty of Pure and Applied Science, University of Cyprus, P.O. Box 20537, Nicosia CY-1678, Cyprus; tarapoulouzi.maria@ucy.ac.cy; 3 Department of Food Science and Technology, University of the Peloponnese, Antikalamos, 24100 Kalamata, Greece; s.agriopoulou@uop.gr; 4 IRCCS CROB, Centro di Riferimento Oncologico della Basilicata, 85028 Rionero in Vulture, Italy; teresa.damore@crob.it

**Keywords:** animal and animal-derived matrices, authenticity, adulteration, chemometric tools, analytical techniques, milk, dairy products, meat and meat products, eggs, fish and fishery products

## Abstract

Food fraud is a matter of major concern as many foods and beverages do not follow their labelling. Because of economic interests, as well as consumers’ health protection, the related topics, food adulteration, counterfeiting, substitution and inaccurate labelling, have become top issues and priorities in food safety and quality. In addition, globalized and complex food supply chains have increased rapidly and contribute to a growing problem affecting local, regional and global food systems. Animal origin food products such as milk, dairy products, meat and meat products, eggs and fish and fishery products are included in the most commonly adulterated food items. In order to prevent unfair competition and protect the rights of consumers, it is vital to detect any kind of adulteration to them. Geographical origin, production methods and farming systems, species identification, processing treatments and the detection of adulterants are among the important authenticity problems for these foods. The existence of accurate and automated analytical techniques in combination with available chemometric tools provides reliable information about adulteration and fraud. Therefore, the purpose of this review is to present the advances made through recent studies in terms of the analytical techniques and chemometric approaches that have been developed to address the authenticity issues in animal origin food products.

## 1. Introduction

In the modern world, the concepts of food safety and quality have expanded. Although they are inextricably linked to the hygiene and wholesomeness of food and, by extension, to consumers’ protection, they also incorporate the knowledge on food fraud and authenticity, intended as the adulteration, counterfeiting, substitution, addition and/or omission of ingredients/information on both the processing and origin of the products [1,2]. On the other hand, the consumer has become increasingly aware and sensitive to the communication/implementation of risk, and to the notions of healthy eating and nutrition as powerful tools to prevent and treat the onset of many diseases [3]. Therefore, the food system has to face the challenge of demonstrating not only the healthiness, nutritional value, safety and quality of the product, but also its sustainability, traceability and authenticity [4]. As a consequence, food scientists, assessors and managers have developed more informative, sensitive and accurate analytical methods to meet these needs.

**Figure 1:**
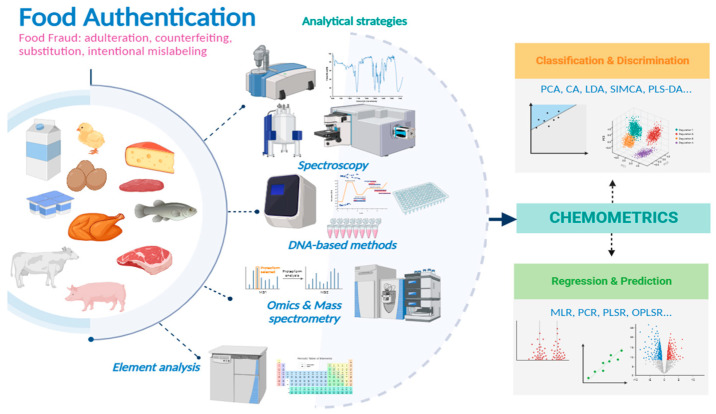
The implementation of authenticity analysis in animal and animal-derived matrices with the help of chemometric tools.

These high-throughput and high-content methods generate a large amount of data, which in order to be best evaluated need to interface with biostatistical, bioinformatic and chemometric approaches. In particular, in the analytical branch of food authentication, complex protocols of sample preparation followed by analytical separation, identification and quantitation are “hyphenated” with advanced chemometrics [5,6]. This interdisciplinary statistical-based discipline is used to identify the relationships between many variables (multivariate analysis), and to analyse, elaborate and interpret the data. The construed models are validated and become able to substantially discriminate between and classify the food products. In these ways, the data generated from conventional analytical techniques, chromatographic, spectrophotometric, biochemical, immunochemical and histological methodologies, and omics applications are complemented [7,8]. Figure 1 presents the implementation of authenticity analysis in animal and animal-derived matrices with the help of chemometric tools.

In this framework, the aim of this review is to give a comprehensive overview of analytical methods used for food authentication purposes, with a special attention on animal and animal-derived matrices, i.e., meat and meat products, milk and dairy products, and fish and fish products. Furthermore, with this study, the authors would like to emphasize the power of chemometric protocols, discussed in depth, used for processing the datasets. These considerations may be a useful tool not only for researchers and researchers, laboratories and stakeholders but also for governments and authorities since the majority of these methods for animal-derived matrices do not meet regulatory acceptance criteria yet [9]. In fact, although the targets of the majority of detected food frauds are fish, honey, dairy products and meat, in the current worldwide legislations, there are few official and internationally accepted methods for these applications. Thus, the authentication of food products has become a major priority and concern not only for safety reasons but also for preventing economic fraud since these matrices are among the most internationally traded food commodities [10,11,12].

## 2. Chemometrics of Milk, Dairy Products, Meat and Meat Products, Eggs, Fish and Fishery Products

### 2.1. General Considerations: Chemometrics and Multivariate Analysis

The animal-derived matrices, discussed in this review, are characterized by very different physicochemical characteristics. Similarly, the topic of food authenticity is complex with many attributes that should be considered and assessed. Environmental and growing conditions, feeding and feed types, and the processing, handling and storage of materials are only some variables that may influence the results of the analytical controls of these matrices. If examined through the usage of classical descriptive statistics, exploring each variable separately (univariate analysis), the results often have no statistical significance and offer a partial image of food origin and global composition. For this reason, in the last decades, researchers and academics have made an effort to give more power to authentication studies by merging them with multivariate analysis. The resulting combination of analytical and chemometric studies is currently the best approach for a precise, global and multiview analysis and to fight and win the battle of food fraud.

### 2.2. Chemometrics of Milk and Dairy Products

Cheese is widely consumed worldwide and represents a well-known food product. New approaches that partially or fully replace milk fat derive from the increasing demand for nutritionally enhanced or functional dairy products from the cheese industry. This has also led to the identification of new methods of adulteration, which may result in these fully/partially substituted products being marketed as cheese [13]. There are over 4000 different types of cheeses worldwide, and dairy fat consists of approximately 400 fatty acids, making it one of the most complex dietary fats [14]. A growing issue in the dairy product market is the incorrect labelling or adulteration of high-value milk samples, such as goat’s or sheep’s milk, with cow’s milk [15]. Adulterations in milk primarily involve water, reconstitution agents (oils, sugar from sugarcane, animal fats), thickening agents (starch, urea, glucose, salt, etc.), preservatives (formaldehyde, sodium carbonate, hydrogen peroxide, etc.), melamine and more [16].

Traditionally, goat’s milk is used in the production of dairy products that have undergone fermentation, especially cheese and yogurt. The nutritional characteristics of goat’s milk (less allergenic protein fraction, higher lipid content and high mineral availability) have increased its demand and production; hence, its high susceptibility to adulteration. Its easy dilution with water and cow’s milk whey (from cheese processing), or even mixing with cow’s milk, gives goat’s milk a higher market value compared to cow’s milk, which is abundant. Hence, the adulteration of goat’s milk with cow’s milk has become more evident nowadays, and this particularly targets many consumers with lactose intolerance and cow’s milk protein allergies, leading to various allergic disorders [17]. Therefore, goat’s or sheep’s milk with cow’s milk added alters the sensory characteristics of the final product and also poses a significant risk to consumers with intolerance or allergies to cow’s milk. Similarly, adulteration with urea in goat’s and cow’s milk often occurs to compensate for protein content [17,18].

An illegal and significant adulteration is the deliberate addition of formaldehyde to raw milk, with the aim of extending the shelf life of milk at room temperature. Rapid deterioration of milk is caused by a high moisture content. Hence, the preservative and antiseptic properties, along with the ability to improve the appearance, including the odour of milk, need to be presented, and this is carried out by formaldehyde. However, it should be considered that formaldehyde is classified as a human carcinogen by the International Agency for Research on Cancer (IARC), being toxic at low concentrations [16,19]. Higher levels of formaldehyde in foods can lead to symptoms such as nausea, coma, abdominal pain, dermatitis, eye irritation, asthma and more [16]. Formaldehyde has been found in milk available in Brazil, Kenya, India and Pakistan, among other places. Another case of adulteration to remember took place in China in 2013 with melamine detection in milk powder. Melamine was added to increase the apparent protein content, leading to dramatic consequences for public health. [19].

The need to include the geographical origin of foods on labels for many commercial products, such as cheeses produced in a specified region with specific physicochemical and sensory characteristics, is depicted in recent EU regulations. Consequently, geographical origin is considered a significant indicator [20]. A usual form of adulteration in dairy product production is the substitution of one type of milk with another due to the lower cost and year-round availability. Some milk products, such as milk powder, are susceptible to dilution or adulteration with exogenous fats or oils. Additionally, the incorrect declaration of geographical origin is another common form of fraud. There is a risk of adulteration due to the violation of PDO protocol specifications in the case of high-value dairy products such as some PDO (Protected Designation of Origin) cheeses.

There is a high demand for the development of portable NIR devices specifically in the production chain of goat dairy products since they are effective in distinguishing between authentic and adulterated samples, for a reduction in the economic cost and analysis time. This will target the production of high-quality products and consumer safety while simultaneously controlling the nutritional value stated on their labels [21].

### 2.3. Chemometrics of Meat and Meat Products

The growing issue of meat fraud occurs in the increasingly globalized and complex food supply chains. In the meat industry, substitution fraud mainly concerns meat derivative products, which consist of minced meat mixed with other ingredients to manufacture sausages, salami, kebabs, burgers, meatballs and stuffed pastas. Raw meat must be correctly labelled when it comes from different animal species, otherwise it becomes fraudulent. When other parts of animals, such as fat, collagen, entrails or internal organs, are added to minced meat during the manufacturing process, fraud might occur [22]. Typical meat products including raw sausages or ham made from game species such as wild goats (chamois), red deer or chamois are consumed in certain alpine regions, such as Switzerland, Austria, Germany and Italy. Game meat is more expensive and easier to substitute with other meats that have a remarkably similar taste, colour and appearance due to its scarcity, coupled with challenges in hunting activities due to increased habitats [23].

The quantity of each ingredient must be declared in meat-derived products, and this is known as quantitative ingredient declaration. The identification of species and differentiation of animal tissues in meat products are of significant concern regarding consumer protection against illegal and/or unwanted adulteration, not only for economic and health reasons but also for religious matters. The presence of pork derivatives such as pork skin, lard, pork meat and pork gelatine in any food products is a significant issue for religions such as Islam, Judaism and Hinduism, which prohibit their followers from consuming pork and its derivatives. Furthermore, halal and kosher, which are still widely practiced by Muslims and Jews, respectively, and certified accordingly, guide the production, slaughter and preparation of meat for human consumption. To be considered kosher, an animal’s meat must come from a cloven-hoofed animal. For example, halal-labelled products should not include the substitution of non-halal meat, and this is considered to be an illegal and unacceptable practice according to Islamic law. Fraud and the adulteration of halal meat products occur due to financial incentives. Food manufacturers in many countries nowadays choose to use pork meat or pork derivatives (pork fat, pork gelatine, etc.) because they are inexpensive and readily available [24,25].

The meat industry faces ongoing challenges in ensuring the authenticity and quality of its products. Fraudulent practices, mislabelling and adulteration are persistent concerns. However, the integration of chemometrics, a multidisciplinary field encompassing chemistry, mathematics and statistics, offers robust solutions to address these issues effectively. This essay explores the diverse applications of chemometrics in safeguarding the authenticity of meat and meat products across numerous studies.

### 2.4. Chemometrics of Eggs

Data interpretation and visualization can be carried out by chemometrics, which provides powerful tools. Principal Component Analysis (PCA) helps in reducing the dimensionality of complex datasets, making it easier to identify patterns and trends in the chemical composition of eggs. This allows researchers to gain a comprehensive understanding of the factors affecting egg authenticity. Chemometric methods enable the clear differentiation between different types of eggs, such as organic and conventional eggs, based on their chemical profiles. By applying multivariate statistical techniques, researchers can establish distinct chemical fingerprints for each egg type, enhancing the ability to detect fraudulent labelling and ensure accurate product labelling.

During the long-term storage of eggs, a significant change that occurs in the egg is the reduction in the elasticity of the vitelline membrane, allowing for easier migration of water from the albumen through the weaker vitelline membrane. The result of this process is that the yolk becomes flatter, and thus, the yolk index parameter measures the thickness and diameter of the yolk. Therefore, the yolk index indicates the viscosity of the yolk, and the higher it is, the better the quality of the egg. In addition, γ-aminobutyric acid was found to be a good marker of the age of eggs during storage [26].

Chemometrics aids in the identification of egg production systems by analysing the chemical composition of egg components. Puertas et al. [27] employed UV-VIS-NIR spectroscopy and chemometric techniques such as SVM, LDA and QDA to identify different egg production systems. Their study focused on the analysis of yolk lipid extracts.

In addition, S-ovalbumin is a biological marker that arises from albumen (the most abundant protein found in eggs). S-ovalbumin is highly correlated with storage time, with low physical variability, and has the potential to become a common indicator for assessing egg freshness [28]. The freshness and storage time of eggs were assessed using chemometric models based on parameters such as the Haugh unit, pH of albumen and yolk height. These parameters were measured using spectroscopic techniques such as VIS-NIR spectroscopy [29]. Moreover, liquid chromatography–tandem mass spectrometry for the quantitation of lipidomic profiles in the yolk granule and yolk plasma of egg yolk was used by He et al. [30]. The recorded differences revealed by using chemometrics, particularly PCA and OPLS-DA, enlighten the need to study new functional and high-value novel egg products.

Furthermore, determination of the geographical origin of eggs can be carried out by chemometric approaches. In addition, chemometrics assists in predicting the freshness and storage time of eggs based on key parameters such as the Haugh unit, pH and yolk height. By establishing mathematical models, chemometrics enables the estimation of egg quality over time, ensuring that consumers receive fresh products. What is more, studies such as that of Joshi et al. [31] have demonstrated the use of chemometric models to distinguish genuine eggs from counterfeit ones based on their chemical properties. This helps in maintaining product integrity and consumer trust. Moreover, by providing accurate and objective assessment tools, chemometrics contributes to quality assurance in the egg industry. Reliable authenticity testing enhances consumer confidence, promotes fair trade practices and ensures that consumers receive the products they pay for.

In conclusion, chemometrics plays a pivotal role in addressing various aspects of egg authenticity, ranging from the differentiation of egg types to determining geographical origin and predicting freshness. Its ability to handle complex chemical data and provide actionable insights makes it an invaluable tool in ensuring the accuracy and reliability of egg quality assessments. By combining various analytical techniques with advanced data analysis methods, several studies have successfully differentiated between different egg types, identified production systems, determined geographical origins, and predicted freshness and storage times. These findings highlight the importance of chemometrics in ensuring the quality and authenticity of egg products, contributing to consumer confidence and fair-trade practices in the egg industry.

### 2.5. Chemometrics of Fish and Fishery Products

The employment of qualitative spectroscopy and chemometrics applied to authenticate fish and seafood products is developed in this section. In fact, several spectroscopic techniques have focused on fish species substitution, geographical origin misrepresentation, and the processing and production method. Using PLS and PCA, Gayo et al. [32] utilized VIS-NIRS to distinguish Atlantic blue crab mixed with the meat of blue swimmer crab. These models are better able to predict the adulteration since the standard errors of prediction (SEP) of 0.252 (PLS) and 0.244 (PCA). NIR spectroscopy reviewed seven species of freshwater fish [33]. In this study, to distinguish between fish samples, PCA, PLS and fast Fourier Transform (FFT), coupled with LDA models, were established by nine preselected spectra wavelengths, and a good prediction of the approved strategy was revealed. The PCA-LDA and FFT-LDA models were performed with high accuracy, specificity, sensitivity and precision. According to Zhang et al. [34], three fish surimi species were categorized by NIR vibration. Using PCA, a disjointed cluster related with red coat surimi species was noticed, and a full distribution rate was provided by LDA findings. Alamprese and Casiraghi [35] developed FT-NIR and FT-MIR to (i) assess the replacement of prized red mullet and plaice species with low-cost Atlantic mullet-and-flounder, and to (ii) distinguish fresh and frozen–thawed fish. These authors noted that LDA and SIMCA associated with FTIR displayed a high difference between samples, and was used to separate fresh and frozen ones. In this sense, a specificity of >95% was determined and sensitivity values were >60%.

Cozzolino et al. [36] used NIR/PLSR to authenticate the fishmeal batches from different fish species. They concluded that dummy PLSR achieved ~80% good classification; in addition, PCA had a score of >80%.

In addition to NIR, further vibrational spectroscopic tools have been extensively promised to be able to perceive fraud in fish and fish products. For instance, MIR was employed to identify fraud by replacing Atlantic salmon with rainbow trout [37]. Using PCA/PLSR, 12 formulations were effectively predicted. Similar trends were observed by Chen et al. [38] who used Raman vibration.

Rašković et al. [39] applied Raman spectroscopy for the classification of 12 different fish fillets of different species. By using HCA/Raman spectra, three separated clusters were revealed. To separate samples, belonging to cod, haddock, saithe and pollack, Standal et al. [40] evaluated and characterized their phospholipid profiles, obtained by ^13^C NMR. Linear analysis contributed a 78% classification rate, while the Bayesian belief network (BBN) achieved a classification equal to 100%. To distinguish between wild/farmed salmon according to the degree of processing, Capuano et al. [41] utilized 1H NMR. These authors reported that by employing SIMCA, a full separation was achieved on the oleic and linoleic acid levels. Similar trends were reported by Vidal et al. [42] who discriminated between farmed/wild European sea bass due to great di-unsaturated acyl groups.

By applying PNN and SVM techniques, Masoum et al. [43] applied the 1H NMR to separate salmon fish oils from eight different geographical sites. These authors noted a grouping % of 98.5 and 100%, respectively. Likewise, promising results were reported by Dalle Zotte et al.’s [44] study that used the combination of ^1^H-NMR/PCA and LDA. These authors noted a perfect separation between wild/farmed samples. In the same way, LDA variables’ selection allowed a classification of 100% of the tested wild and farmed samples. Protein structures, exaggerated by thermal processing, have been examined by the application of spectroscopic tools. He et al. [45] investigated the impact of wet cooking on the myosin/ctin denaturation in false abalone by the heat transfer model. These authors concluded that immobilized water was condensed with a prolonged processing time, and the shear force was also reduced by LF-NMR and MRI. PLSR showed a great relationship among immobilized water and sensory studied features. By fluorescence microscopy joined with physicochemical changes, Cropotova et al. [46] measured the lipid oxidation in sous-vide-cooked Atlantic mackerel. At 70 °C and 80 °C during 10/20 min it was achieved with/without antioxidants. Fluorescence micrographs of extracted lipids were acquired in λ_ex_ 475/40 and λ_em_ 530/50, and interrelated with TBARS. It was stated that the conjugated trienes produced by lipid polymerization throughout the storage of processed mackerel were associated and correlated with the instrumental yellowness of the fish flesh. Fluorescence at 415 nm and 347 nm for uncooked and cooked fish fillets was assessed by Tavares et al. [47], and the outcomes showed the highest intensity was observed in baked and fried samples compared to the raw and boiled ones. Xia et al. [48] employed NMR and MRI to investigate the boiling, frying and stewing of turbot. Assessments were connected with texture and colour quality. A good separation was made between cooking methods and was shown by PCA/NMR data. Moreover, NMR results were confirmed by weighted images of the MRI scans with exposed conceptions of interior structural data. Some modern spectroscopic tools for tracking thermal handlings in fish and fish products are outlined in Table 1. 

## 3. Current Analytical Methods for Milk, Dairy Products, Meat and Meat Products, Eggs, Fish and Fishery Products Authentication and Chemometrics

### 3.1. Overview of Analytical Methods

As shown in Figure 1, the analytical techniques used for food authentication protocols are very diverse. Each technique can have different configurations, applications, advantages and disadvantages.

The spectroscopic techniques, although extremely diversified, are united by the principle of the emission or absorption of electromagnetic radiation with the consequent generation of continuous spectra, band spectra or line spectra. Each substance is capable of emitting or absorbing particular radiations with intensity dependent on the concentration of the substance itself. Among the most used are Nuclear Magnetic Resonance spectroscopy, Infrared and Near-Infrared spectroscopy, and Raman spectroscopy, based, respectively, on the measurement of paramagnetic spin transitions, vibrational transitions and the inelastic scattering of photons. These techniques are able to provide a fingerprint of the substance and are particularly useful for geographical traceability studies of food products.

Similarly, mass spectrometry, which can be used for both inorganic and organic and hyphenate analysis with different types of chromatographic separation, can provide the characterization, identification and precise and sensitive quantification of all small molecules, metabolites, macromolecules and trace elements, which have ionizability as their characteristic.

Finally, to establish the species and the genomic and transcriptomic characteristics, the techniques based on DNA analysis, which today have found their maximum use in the next generation sequencing (NGS) techniques preceded by the construction of complex libraries, are today the most studied and investigated.

### 3.2. Authenticity of Feed Materials towards Egg Authenticity

Determination of the geographical origin of food products is a crucial step; however, it might not be sufficient on its own to guarantee authenticity. Mapping and controlling the entire supply chain, including raw feed materials, could be essential for precise control over the various factors influencing their quality and authenticity.

For example, diet variations among hens even within the same geographical area might introduce complexities in ensuring consistent quality and authenticity. Mapping the raw feed materials becomes crucial in ensuring precise control over various aspects impacting egg authenticity. It is not just the location where hens are raised that matters but also the source and quality of their feed. Differences in feed can significantly affect the nutritional composition and even the chemical markers present in eggs.

Formulation of an effective feed should consider the right cost and nutritional quality to cover the essentials of poultry as reported by Belkhanchi et al. [54].

For livestock, the feed must also provide enough nutrients to meet production needs (eggs or meat). Different forms can comprise the feed such as: raw materials, compound feed (a mixture of at least two raw materials), complete feed (compound feed with sufficient composition, to cover the daily requirements) or supplementary feed (for example, cereals to supplement the raw materials given to the animal) [55,56].

The increase in the needs of poultry arises from the growth of global demand for animal protein [57], thus creating many challenges [58,59,60,61,62,63,64,65]. Tremendous changes in the growth of all phases have been historically observed for the commercial poultry industry from the hatchery to broiler and layer farm practices across meat and egg processing technological advances [66]. Hence, the volume of poultry meat and eggs produced has also expanded to match this rise in retail and consumer demand [67,68,69]. This rise still depends on advances in bird genetics, nutritional management, processing technologies and food safety [70,71,72,73,74,75,76].

The success of a quality feed formulation depends on the physicochemical characteristics of raw materials [77] and the production efficiency and meat quality in broiler chickens derived from the effect of the partial replacement of raw materials with others [78,79,80,81,82].

According to the European Union (EU)-funded project MARLON, the organization and characteristics of specific livestock and feed production chains (conventional, organic, GM-free) within the EU, with an emphasis on controls, regulations, traceability and common production practices, have been studied. Moreover, the origin of animal feed used in the EU as well as an examination of the use of genetically modified organisms (GMOs) in feed is provided according to Kleter et al. [83]. They showed that livestock is traceable at the herd or individual level, depending on the species. Geography and animal species affect husbandry practices, which can vary. For feeds, only coarse estimates could be made for the amount of GM feed ingredients that an animal is exposed to.

The approach followed by EU risk assessors is described in a detailed guidance developed by the European Food Safety Authority’s panel of experts on genetically modified organisms (EFSA GMO Panel) and incorporated into EU legislation [84,85].

The authenticity of native eggs was detected by combining near-infrared (NIR) spectroscopy with data-driven-based class modelling (DDCM) and model-independent variable selection, i.e., joint mutual information (JMI) as reported by Chen et al. [86]. A total of 122 eggs of three types were collected. Principal Component Analysis (PCA) was utilized for exploratory analysis. Near-infrared (NIR) spectroscopy has become increasingly important in food field as a powerful analytical technique [87,88,89,90,91,92]. NIR spectroscopy can characterize multiple chemical components of samples showing great advantages such as A lower sample preparation requirement, reduced analysis time and cost, multicomponent analysis and the potential for online analysis. NIR spectral information is hardly selective due to the NIR spectrum corresponding to overtones and combinations of the fundamental molecular vibrations. NIR-based quantitative and qualitative analyses need the help of chemometrics.

Another study by Rogers et al. [93] used stable isotopes to develop authentication criteria for eggs laid under cage, barn, free range and organic farming regimens from the Netherlands and New Zealand. Commercial poultry feeds and egg albumen from 49 poultry farms across the Netherlands were used to determine the isotopic variability in organic and conventional feeds. Trophic effects of these corresponding feeds and barn, free range and organic farming regimens on corresponding egg albumen were also assessed. This study suggested that nitrogen showed particular promise as a screening and authentication tool for organically farmed eggs. They proposed that Dutch organic egg whites should have a minimum δ^15^N value of 4.8‰ to account for an organic plant-derived diet. Regarding New Zealand egg isotopes over the past 7 years suggested that organic eggs should have a minimum δ^15^N value of 6.0‰, a higher value due to the use of fishmeal or meat and bone meal (MBM), restricted in the EU.

Finally, Bandoniene et al. [94] developed a method for labelling poultry products by the selective enrichment of two rare earth elements (REE), namely, terbium and thulium, in the feed for laying hens to discriminate labelled from unlabelled poultry products. Analysis was varied by using either conventional or laser ablation inductively coupled plasma mass spectrometry. This was found to be a good methodology to detect authenticity.

### 3.3. Current Analytical Methods for Milk and Dairy Products Authentication and Chemometrics

Li et al. [95] as well as Huang et al. [96] noted that Raman spectroscopy is gaining more and more attention in food quality control in combination with chemometrics due to it being fast, portable and non-destructive. Furthermore, this technology allows the measurement of intact samples (while inside the packaging), while the water content of the samples does not affect measurements.

One of the primary applications of chemometrics in the dairy industry is the detection of adulteration. Numerous studies in the provided table demonstrate how chemometric techniques can effectively identify various forms of adulteration, including the addition of water, non-dairy substances and contaminants such as melamine. For example, FT-NIR spectroscopy combined with chemometric methods such as PCA, PLS-DA and iPLS was employed by da Paixao Teixeira et al. [21] to detect the adulteration of yogurt and cheese made with goat’s milk using bovine milk. Such techniques provide rapid and accurate detection, bolstering consumer trust and safety. Differentiation of dairy products based on their geographic and seasonal origin can be carried out by chemometrics, and this is particularly valuable for products with Protected Designation of Origin (PDO) status. Studies such as Pellegrino et al. [97] and Tarapoulouzi and Theocharis [98] employed chemometric methods to distinguish between different types of cheese. This verification is vital for protecting traditional cheese-making practices and ensuring consumers receive genuine products. More recently, Tarapoulouzi and Theocharis [99] discriminated Halloumi and Anari cheese in two classes, thus per cheese type. In addition, they discriminated samples based on milk species, i.e., cow and goat–sheep origins for each cheese type. They combined Fourier Transform Infrared (FTIR) spectroscopy with Orthogonal Partial Least Squares Discriminant Analysis (OPLS-DA). The success of this study highlighted the importance of FTIR spectroscopy in combination with chemometrics in food authenticity. Furthermore, beyond authenticity, chemometrics aids in quality control and process verification. It facilitates the monitoring of dairy product quality by analysing various chemical and physical parameters. For instance, using rheology and FT-NIR spectroscopy, combined with chemometric analysis, Strani et al. [100] assessed the impact of physicochemical parameters and the use of skimmed milk powder in milk thickening. This approach enables producers to maintain product consistency and quality.

The authenticity of milk and dairy products is ensured by the pivotal role played by chemometrics applications. They serve as powerful tools for detecting adulteration, verifying the origin of products, identifying species, confirming cheese types and maintaining product quality. These applications not only enhance consumer confidence but also help protect the integrity of traditional dairy products and support quality control efforts within the dairy industry. As technology continues to advance, the role of chemometrics in the dairy sector is poised to become even more critical, guaranteeing that consumers receive authentic and high-quality dairy products. Table 2 presents recent studies of chemometrics and the authenticity of milk and dairy products. 

### 3.4. Current Analytical Methods for Meat and Meat Products Authentication and Chemometrics

Khan et al. [118] used Fourier Transform Infrared Spectroscopy (FTIR) along with Principal Component Analysis (PCA) and Partial Least Squares Regression (PLSR), combined with chemometrics, enabling the analysis of a complex multitype of meat blends. This approach ensures the maintenance of desired physicochemical characteristics in beef, pork, chicken and turkey meat products. This chemometric approach ensures the reliable identification of adulterants, enhancing product quality and consumer trust.

Varrà et al. [119] monitored the impact of radiation processing on sausages. NIR spectroscopy, employing the chemometric tools of Principal Component Analysis (PCA) and Orthogonal Partial Least Squares-Discriminant Analysis (OPLS-DA) provided the necessary insights to maintain product safety and quality. Achata et al. [120] monitored bacterial growth in beef muscle under various storage conditions, which is a critical study for food safety. They used Vis-NIR hyperspectral imaging along with the total viable count (TVC) and Partial Least Squares Regression (PLSR). Rebellato et al. [121] worked to determine the sodium content in hamburgers by applying Near-Infrared spectroscopy (NIR). Chemometrics, including Partial Least Squares (PLS) and PLS Discriminant Analysis (PLS-DA), supported this assessment effectively. Leng et al. [122] identified adulteration with pork and duck meat in ground beef products. NIR spectroscopy, in conjunction with Discriminant Analysis and Partial Least Squares (PLS), helped to achieve this goal. A very recent study performed by Cozzolino et al. [123] took place to differentiate between traditional and wild meat species. PCA, LDA and the similarity index (SI) were applied. The authors concluded that SI is a quite simple method for comparing two spectra. Comparison of SI to classical chemometric methods (e.g., LDA, PCA), showed that SI can be more easily understood, has a low cost and can be applied by only using software such as Excel^®^ (2013). Another study based on NIR spectroscopy was implemented by Hoffman et al. [124]. The adulteration of exotic meat species (emu and camel) with traditional or commercial species (beef and lamb) in binary mixtures of minced meat was monitored by chemometric methods, such as PCA and PLS-DA. It was concluded that the level or ratio of adulteration can be determined by NIR spectroscopy.

Based on the aforementioned research studies, chemometrics stands as an indispensable asset in the realm of meat and meat product authenticity studies. Its capabilities span from precise adulteration detection to the characterization of multi-meat blends, authentication of halal products, differentiation of meat types, monitoring of processing effects, and assessment of biochemical and physicochemical properties. Furthermore, chemometrics plays a pivotal role in food safety by monitoring microbial flora and ensuring compliance with dietary recommendations. By embracing chemometrics, the meat industry can ensure that consumers receive products of the utmost authenticity and quality. Table 3 presents recent studies of chemometrics and the authenticity of meat and meat products.

### 3.5. Current Analytical Methods for Fish and Fishery Products Authentication and Chemometrics

#### 3.5.1. DNA-Based Methods

In order to identify fish species, several DNA-based methods have been employed. Their realization involved multiple similar preparative steps, viz. the isolation of DNA and in silico investigation applying convenient databases (such as specific primers). The most noteworthy for fish species identification are methods using restriction cleavage RFLP and AFLP (Amplified Fragment Length Polymorphism), DNA barcoding, FINS (Forensically Informative Nucleotide Sequencing), HRM (High-Resolution Melting), PCR (Polymerase Chain Reaction), RAPD (Random Amplified Polymorphic DNA) and SSCP (Single-Stranded Conformational Polymorphism) [137,138]. In addition, the Loop-Mediated Isothermal Amplification (LAMP) technique has been newly employed for fish species identification. Several methodologies have been carried out using either nuclear DNA (nDNA) or mitochondrial DNA (mtDNA), but recently, LAMP (Loop-mediated isothermal amplification) method has been applied for fish species [139,140]. Nuclear (nDNA) or mitochondrial mtDNA have been used in several lines. Databases of genome sequences/nucleic acid sequences could simplify the choice of appropriate target molecules; on the other hand, identification markers can support these methodologies through their complexity and efficiency.

The most important ones are: EMBL (European Molecular Biology Laboratory, http://www.ebi.ac.uk; DDBJ (DNA Data Bank of Japan, http://www.ddbj.nig.ac.jp; and the NCBI (Center for Biotechnology Information, http://www.ncbi.nlm.nih.gov. The EU database FishTrace (https://fishtrace.jrc.ec.europa.eu/) employs databases based on fish nucleotide sequences. Recently, the development of DNA-based identification methods has been shortened by the recognition of the fish genome sequence. For instance, up to 2020, more than 900 whole-genome sequences of fish species were published [141]. mtDNA sequence data can also be employed to identify several fish species in concurrence with the whole-genome sequences, and >3300 mtDNA sequences of fish species have been put in the NCBI. On the other hand, for fish species identification, mtDNA can be used to elucidate the difference in nearly linked taxa and even species, and its aptitude to distinguish the geographical origin of the fish species [142]. According to the literature, for fish species identification, the commonest mitochondrial markers are: the COI (gene for cytochrome-c-oxidase subunit I) [143,144] and cytb (cytochrome b) [145]. Their incidence is expected in treated fish products based on the very high mtDNA copy number in the cell [146]. The non-feasibility of DNA computing, since mtDNA levels could vary on the tissue type, is the major drawback of mitochondrial markers compared to nuclear ones [147]. In addition, β-actin is the most regularly used nuclear marker for fish authentication, and is also employed as an internal regulator for mRNA quantification [148] and parvalbumin [149]. Additional regularly used markers for fish species identification are: short tandem repeats (STRs) and simple sequence repeats (SSR) [150]. These nuclear microsatellites have the benefits of being extremely species-specific and being utilized for analyses at the intra-species level; meanwhile, their abundance across the genome requires a small amount of DNA to assemble data [151].

The PCR-RFLP has been fruitfully useful for fish species and their products’ identification due to its ease and low cost. AFLP progresses the species-specific SCAR (sequence characterized amplified region) marker for detection of the fresh Atlantic salmon’s adulteration against the less expensive rainbow trout [149]. Maldini et al. [150] applied AFLP to assess the distinctiveness of fish species in processed commercial products, while this approach could quickly classify nine species of cod fish as concluded by Akasaki et al. [151]. Espiñeira et al. [152] used PCR-RFLP to distinguish seven species of anglerfish, and Yu and Guo [153] also appraised the genetic difference of four strains and one wild population of the eastern oyster by AFLP/microsatellite markers. Lin and Hwang [154] utilized PCR-RFLP, and identified the species in eighteen commercial canned tuna products by differentiating albacore, yellowfin, bigeye and Atlantic bluefin tuna. Recently, the investigation of Yao et al. [155] established and validated 39 commercial tuna sashimi samples. RFLP is advantageous due to it being precise, reliable, operative, moderately polymorphic, with great genomic richness and with random dissemination, and extremely reproducible. However, it has some disadvantages such as being expansive, not entirely robotic, and demanding large quantities of purified and great molecular weight DNA for each digestion.

FINS uses a comparable standard to DNA barcoding and is allied with DNA sequencing/phylogenetic analyses. An informative nucleotide sequence is subsequently identified by phylogenetic analysis succeeding the sequencing of an amplified specific DNA fragment [154]. Remarkably, FINS allowed the detection of new unexplored species [155]. For instance, the adulteration of 40% of processed Cyprinidae commercial samples by Oreochromis spp. was noticed using the FINS method [156]. FINS was suitable for extremely treated products and powerful when employed to define the interspecific/intraspecific variability. However, it is expensive, and highly skilled operators are required. Genetic markers employed to identify fish species are summarized in Table 4.

#### 3.5.2. Novel Detection Methods

Recently, novel techniques for assessing fish and fishery products’ features have resulted in the progress of non-invasive/non-destructive instrumental methods, such as biosensors and e-sensors.

##### Biosensor Techniques

Biosensors are able to appraise a biological or chemical reaction and to adapt the answer into an electrical signal [171]. Tang et al. [172] concluded that a sensitive amperometric sensor, based on carbon nanofibers, has a great affinity to the oxidation of Xanthine in crucian carp samples. The correlation coefficient and the limit of detection were 0.99 and 20 nM, respectively. Heising et al. [173] used electrode sensors to monitor pH variation in and conductivity of the aqueous phase, which was associated with the increase in volatile amines in fish. From raw fish flesh, Chang et al. [174] established an amine gas sensor qualified to perceive volatile amine gases such as ammonia, TMA and DMA. Li et al. [175] reviewed the impact of different triethylamine concentrations on copper nitrate–benzenetricarboxylic acid (Cu-BTC) on the concurrent assessment of Xa and HxA in fish samples. The results showed that TMA had a great influence on the electron transfer ability of Cu-BTC, improving the sensibility. A linear performance between the levels and the oxidation peak of Xa and HxA was performed on fish samples. Enzymatic biosensors, based on the evaluation of the response substance/enzyme, were created. Thandavan et al. [176] found a biosensor based on nanoparticles of iron oxide linked with Xanthine oxidase (XOD), provoking the electroreduction of H_2_O_2_. Narang et al. [177] and Borisova et al. [178] launched a system-based nanocomposite of TiO_2_ nanoparticles/carbon nanotubes and [polydopamine/platinum, which immobilized the enzyme of XOD. In these studies, labeo and hake fish samples were examined, and these novel biosensors proved to have high reproducibility and repeatability. Similarly, Apetrei et al. [179] proposed a sensor for histamine biosensing, where the enzyme diamine oxidase was immobilized on a carbon blended with grapheme/platinum nanoparticles. The LOD was equal to 25 mM at a linear array between 0.1 and 300 µM.

Torre et al. [180] developed a biosensor based on a carbon electrode with the immobilized diamine oxidase in tuna and mackerel samples. The LOD was low (0.97 mg/L) and the accuracy, as well as recovery value were high [180,181]. To define HXa, Xa and uric acid in four different fish species, Pierini et al. [182] proposed an edge plane pyrolytic graphite electrode with immobilized nucleoside phosphorylase and XOD. Likewise, to define ATP-related compounds, alkaline phosphatase and adenosine deaminase were concurrently co-immobilized onto alkylamine glass beads [183].

###### Sensory Bionics Technology

This technique comprises an electronic nose/tongue, a colorimetric sensor array and computer vision. In fish products, this technology was developed based on the senses of smell, taste and vision.

- *E-nose*

Numerous systems have been employed based on electrochemical gas, metal oxide and conducting polymer sensors joined with various extraction/data processing methods.

Guohua et al. [184] launched an electronic nose system that consisted of eight metal oxides for envisaging the freshness of grass carp. These authors employed PCA to separate all samples according to the freshness; so, with the storage time, the response intensities of sensors increased. In this study, all samples were separated into three different groups. To discriminate channel catfish according to a good/or not flavoursome aroma, Wilson et al. (2013) applied an e-nose covering an organic matrix-coated polymer-type 32-sensor array. To track the spoilage of tilapia, Semeano et al. [185] recently developed an optical electronic nose linked to the microorganism’s growth. Haugen et al. [186] monitored the smoked salmon process using a gas-sensor array system. The total viable content (TVC) and lactic acid bacteria (LAB) loads were controlled, and a precise classification rate ranging from 93 to 95% was achieved. In the same way, TVC was detected by four metal oxide microsensors to separate sardines according to freshness [187]. Tian et al. [188] used metal oxide sensors to assess TVB-N and TVC in hairtail fish, and PCA was employed for the compensation of humidity and temperature effects. All these established results prove that e-noses have many benefits in monitoring the authenticity of fish freshness including their ease of operation, rapidity, reliability and precision, which could substitute for other expensive or time-consuming analysis techniques.

- *E-tongues*

To evaluate sea bream freshness, Gil et al. [189] developed a method based on electrodes of several kinds of metals. An artificial neural network was conducted to classify samples; the correct % was equal to 90. Barat et al. [190] employed an e-tongue composed of gold/silver wires. A high correlation was obtained between the sum of inosine + HXa and the sum of all other ATP breakdown products. Miao et al. [191] applied a combination of the e-nose and -tongue for a post-cooking sensory evaluation of canned tuna. A PCA was employed for the creation of a K-value calibration model, gaining an acceptable distribution of the samples by the first two principal components. Pattarapon et al. [191] used an e-tongue, in conjunction with an e-nose and several chemical parameters, to investigate the variations in grass carp quality between vacuum and non-vacuum packing. The outcomes showed that these techs could distinguish between the three different storage conditions, sustained by PCA/LDA analysis [191,192].

- *Computer vision technique*

This technique has been employed to attain and examine the image of a real scene using computers. It has the potential to track the quality of food products in an automated and non-destructive way [193,194]. To assess the colour variations in the pupils and gills of tilapia, Shi et al. [195] used a computer vision system, and the MLR was investigated to predict TVB-N, TVC and TBA values with high R2 (~0.999). Similar trends were observed by Balaban and Alçiçek [196] and Issac et al. [197], who studied fish of gilthead sea bream, and gills of Indian rohu, respectively. It should be noted that, compared with traditional evaluation approaches, computer vision displays the capability to be speedy and has a low-cost for envisaging the freshness and authenticity of fish and fish products.

## 4. Challenge and Future Perspectives

In spite of extended investigations focused on the detection of fraud and authenticity via on-site and real-time advances, numerous pivotal challenges continue to prevail regarding both technique concerns and the framework for the validation of models. In this line, several approaches comprise non-targeted methods that detect diverse, small modifications in the studied food product; furthermore, these extracted data were investigated by a multivariate statistic approach. In this regard, it should be noted that the authentication of food samples should define the unit/number and variability in samples, and a sustained maintenance of the food database is required to guarantee its long-term ability to provide suitable results. On the other hand, various experimental conditions can impact the acquired results and contribute to analytical deviation that is not linked to the authentication issue. These deviations should be decreased to the lowest values and be well monitored to guarantee that they do not confuse the results of the analysis.

In spectroscopic techs, the results of which are reproducible and just influenced by variations in sensitivity, sample development and preparation was not normally required. This is true for liquid foods, whereas solids (viz. meat, fish, egg) are heterogeneous matrixes and may involve moderate and/or multiple preparations. Furthermore, the choice of a proper acquisition mode was crucial to attain authentic spectroscopic results. Based on the food product’s nature, the kind of radiation, the type of sample performance, the versatile sample holder and the employed temperature should be tracked.

Recently, hyperspectral imaging tools have been an effective and useful alternative to point spectral scanning. In heterogeneous food product samples such as meat and fish products, hyperspectral imaging can control the large spatial distribution of components. In these two kinds of animal products, hyperspectral imaging technologies are linked to NIR radiation spectroscopy to determine the quality and the corresponding authenticity.

Generally, from any developed detection system result, multivariate data analysis is the ultimate phase that is skilled at categorizing samples as authentic or not. In this situation, a suitable algorithm and powerful validation of the model are needed to assure reproducible results that favour the agreement of these practices in legislation. Practically, several works have been established and developed at the laboratory scale, while few have been conducted at the plant level, which continues to be challenging. Thus, to meet these challenges, a collaborative effort of all actors involved, including regulatory agencies, industries, stakeholders, academics and researchers, is undoubtedly needed.

## 5. Conclusions

Analytical techniques together with chemometrics are undoubtedly reliable techniques for predicting fraud and the authenticity of animal and animal-derived foods. It is important to find in each case a rapid and non-invasive method, together with the appropriate model of chemometric processing and validation, in order to extract a reliable tool for the rapid identification and quantification of adulteration. This is an important requirement at the current time due to the increasing number of processed animal products in which the treatments applied can cover a possible adulteration between species, against the rights of consumers. A high number of analysed samples and validation systems are needed to demonstrate that the models can accurately predict not only adulteration levels but also simple adulteration identification.

## Figures and Tables

**Table 1:** Some recent examples of spectroscopic techniques used for monitoring thermal treatments in fish and fish products.

Genus/Species of Fish	Applied Technique/Wavelength Range	Model	Main Findings	References
False abalone (*Volutharpa ampullacea perryi*)	NMR, MRI/21.3 MHz	PLSR	Quantitative descriptions of actin and myosin protein denaturation and water distribution	[45]
Atlantic mackerel	Fluorescence: Excitation = 475 nm Emission = 530 nm	Univariate analysis	-Fluorescence/lipid oxidation products are highly correlated	[46]
Alaska pollock surimi	FTIR/4000–400 cm^−1^	PCA	The reduction in the gel strength was consequentce of modifications in protein secondary structures	[49]
Atlantic salmon	FTIR/4000–400 cm^−1^	PCA	Cooking/Electrolyzed water: -significantly reduced *Listeria monocytogenes* -developed protein denaturation	[50]
Bighead carp (*Aristichthys nobili*s)	Raman/400–3500 cm^−1^ and 22.6 MHz	PCA	-A decline in α-helix structures -With the increase in heat treatment, a modification in myosin secondary structures	[51]
Hairtail (*Thichiurus lepturus*) fillets	Fluorescence: Excitation = 347 nm Emission = 415 nm	Univariate analysis	-In cooked fish, an increase in fluorescence was observed -As compared to boiled ones, more fluorescence was noted from baked and fried fillets	[47]
Sturgeon (*Acipenser gueldenstaedtii*)	Fluorescence: Excitation = 360 nm Emission = 380–600 nm	Univariate analysis	-Compared to fluorescence of samples before digestion, the fluorescence was increased after digestion -With roasting times, changes in spectral patters (shape and intensity) were observed	[52]
Turbot	NMR, MRI/21.2 MHz	PCA	Correlation between NMR relaxation parameters/texture and colour measurements	[52]
Atlantic salmon	FTIR/4000–400 cm^−1^	PCA	With high cooking temperature and cooking time, the amid I region exposed an increase in aggregation and protein denaturation	[53]
Fish cakes	NIR/760–1040 nm	PLSR	-In fish cakes, prediction core temperatures were 2.3 °C and 4.5 °C for NIR point system and imaging system, respectively -In the NIR system, T° changes till 11–13 mm depth in fish cakes	[45]

**Table 2:** Recent studies of chemometrics and authenticity of milk and dairy products.

Type of Matrix	Purpose of Study	Method of Analysis	Chemometric Method	References
Goat milk	Detection of water, urea, cow’s whey, cow’s milk	NIRS	PCA, k-NN, PLS-DA, SIMCA	[17]
Fresh milk	Formaldehyde detection	TD-NMR	PCA, PLS, SIMCA	[19]
Fresh cow’s milk	Formaldehyde detection	ATR-FTIR	PCA, SIMCA, PLS, PCR	[16]
Milk coagulation using rennet	Effect of physicochemical parameters and use of skimmed milk powder	Rheology, FT-NIR	MCR-ALS	[100]
Skimmed milk powder	Detection of vegetable protein powder, whey powder, starch, lactose, glucose, fructose as well as non-protein nitrogen such as ammonium chloride, ammonium nitrate, melamine and urea	Multiple optical sensors (UV-Vis, fluorescence and NIRS)	Algorithm οne class classification	[18]
Milk	Milk powder detection	UPLC-QTOF-MS	PCA	[101]
Milk	Geographical and seasonal origin	IRMS, EDXRF, ICP-MS	OPLS-DA, SIMCA	[102]
Milk	Discrimination based on seasonal and animal origin	IRMS	One-way ANOVA	[103]
Fresh milk	Geographical origin	IRMS	PCA	[104]
Milk	Geographical origin	IRMS	OPLS-DA	[105]
Milk and halloumi cheese	Discrimination based on animal origin	FTIR	PCA, OPLS-DA	[106]
Milk	Formaldehyde detection	ATR-FTIR	PCA, SIMCA, PLSR, PCR	[16]
Milk	Detection and quantification of skimmed milk powder in fresh milk	FE-SEM, cyclic voltammetry	PCA, SIMCA, PLS	[107]
Milk and mature cheese	Control of breeding system (agricultural production system) of animals	FTIR, GC, PTR-ToF-MS, sensor analysis	LDA	[108]
White cheese	Adulteration with vegetable fats	Spectroscopy Raman	PLS-DA, PLS	[13]
PDO-Fontina cheese and traditional Fontal cheese	Discrimination according to the type of cheese	GC-IMS, CZE, chromatography	PCA	[97]
Graviere cheese	Geographical origin	GC-MS, ICP-OES	LDA	[20]
Fresh and pasteurized milk	Detection of animal origin and heat treatment	Spectroscopy Raman	PLS-DA	[15]
Cheese	Study of ripening and ripening type based on fatty acid content	Gravimetric GC-MS, Ag^+^-HPLC-DAD	CA, PCA, LDA	[14]
Yogurt and cheese made with goat’s milk	Detection of cow’s milk adulteration	FT-NIR	PCA, PLS-DA, iPLS	[109]
PDO grated cheese Parmigiano Reggiano	Study of authenticity based on crust percentage (maximum allowed rind content percentage)	Spectroscopy Raman	SIMCA PLS	[110]
Cheese PDO-Pecorino Romano, PDO-Pecorino Sardo and Pecorino di Farindola	Discrimination of the type of cheese based on volatile compounds	HS-SPME-GC-MS	PCA, LDA, PLS-DA	[111]
Kefalotyri and cheddar cheese	Discrimination by type of cheese	^1^H-NMR, FTIR	OPLS-DA	[112]
Dairy products	Detection of adulteration with vegetable fats	^1^H-NMR	Orthogonal projection	[113]
Yogurt	Detection of adulteration with vegetable fats	FT-NIR, FT-MIR	SIMCA, PLSR	[114]
Milk powder	Milk powder	ATR-FTIR	PCA, SIMCA	[115]
Classification of Halloumi and Anari cheese	Discrimination according to the origin of the cheeses	FTIR	OPLS SIMCA	[99]
Halloumi cheese, Kefalotyri and cheddar	Discrimination according to the type of cheese	FTIR, ^1^H-NMR	OPLS-DA, MOCA	[116]
Prato and mozzarella cheeses	Verification the authenticity of commercial samples of prato cheese	MIR	PLS-DA	[117]

**Table 3:** Recent studies of chemometrics and authenticity of and meat and meat products.

Type of Matrix	Purpose of Study	Method of Analysis	Chemometric Method	References
Chicken	Status identification: fresh or frozen	NIR	PLS-DA, CPANN, SVM	[125]
Sausages (pork and beef mix)	Soy protein adulteration	E-nose	PCA, PNN	[126]
Beef meat preparation	Wild boar meat adulteration	FTIR	PCA, PLS	[127]
Beef, lamb and venison	Discrimination red meats	Spectroscopy Raman	PCA, PLS-DA και SVM	[128]
Beef mixture	Chicken adulteration	ATR- FTIR	PCA, PLSR, ANN	[129]
Beef meat preparation	Adulteration with another type of meat	FTIR	PCA, PLS-DA και SIMCA	[130]
Minced pork	Aging and wear, characterization of changes during storage and spoilage	HS-SPME-GC-MS	PCA, OPLS-DA	[131]
Beef meat	Adulteration with maltodextrin	MIR	PLS-DA	[132]
Beef, pork, chicken and turkey meat	Physicochemical characteristics	FTIR	PCA, PLSR	[118]
Sausages	Radiation treatment process	NIR	PCA, OPLS-DA	[119]
Beef muscle	Bacterial growth at two storage temperatures	Vis-NIR HIS	TVC, PLSR	[120]
Hamburger	Sodium content	NIR	PLS, PLS-DA	[121]
Ground beef	Adulteration with pork and duck meat	NIR	DA, PLS	[122]
Beef sausage	Adulteration with pork	LC–HRMS	PLS-DA	[133]
Sausage products	Adulteration with pork	GC-MS, FTIR	PCA	[134]
Pork meat	Authenticity of pork fat according to the rearing system	NIR	DD-SIMCA	[135]
Chicken	Geographical origin	ICP-OES, ICP-MS	OPLS-DA, CDA	[136]

**Table 4:** Recent overview of genetic markers employed for fish species identification.

Genus/Species of Fish	Detection Method	Markers	Objective of Study	References
Anglerfish (Lophius)	real-time PCR	Pvb/nDNA	Identification and quantitation of two European anglerfish, *L. piscatorius* and *L. budegassa*	[157]
Salmon and trout	real-time LAMP	cytb/mtDNA	Individually and simultaneously specific detection of *S. salar* and *O. mykiss*	[145]
Salmon and trout	real-time PCR	COI/cytb mtDNA	Identification of *S. salar* and *O. mykiss* in processed fish products	[158]
Atlantic salmon (*Salmo salar*)	real-time LAMP, PCR	Cytb/mtDNA	Detection of *S. salar* in processed fish products	[159]
Sardina	DNA barcoding, real-time PCR	COI/mtDNA	Screen the species of *S. pilchardus* species in varied products	[160]
Codfish species	HRM	12S rRNA/mtDNA	Discrimination between Gadus species and the other five Gadiformes species. Nineteen commercial codfish products were included in the Gadus cluster, cross-confirmed by the real-time PCR and DNA barcoding	[161]
Salmon	LAMP, PCR	cytb (LAMP), COI (PCR)/mtDNA	Identification of Atlantic salmon in processed fish product	[162,163,164,165]
Trout	PCR, LAMP	COI (PCR)/ cytb (LAMP) mtDNA	Identification of rainbow trout in processed fish products	[163,164,165,166]
Salmon	real-time PCR	GH1, 18S rDNA/nDNA	Detection of *S. salar* in processed fish products	[167]
Fish genus	real-time PCR	16S rRNA/mtDNA	Validation and applicability to model mixtures with spiked fish	[168]
Salmon	SNPs 94	SNPs loci/ Genome	Discriminate between farmed salmon populations of several origins	[169]
Sardina	PCR-RFLP	Cytb/mtDNA	The authenticity of sardines commercialized in Rio de Janeiro state	[170]

## References

[1] Gallo M., Ferranti P. (2016). The evolution of analytical chemistry methods in foodomics. J. Chromatogr. A.

[2] González-Domínguez R. (2020). Food Authentication: Techniques, Trends and Emerging Approaches. Foods.

[3] Kendall H., Clark B., Rhymer C., Kuznesof S., Hajslova J., Tomaniová M., Brereton P., Frewer L. (2019). A systematic review of consumer perceptions of food and authenticity: A European perspective. Trends Food Sci. Technol..

[4] Medina S., Perestrelo R., Silva P., Pereira J., Câmara J. (2019). Current trends and recent advances on food authenticity technologies and chemometric approaches. Trends Food Sci. Technol..

[5] González-Domínguez R., Sayago A., Fernández-Recamales Á. (2022). An Overview on the Application of Chemometrics Tools in Food Authenticity and Traceability. Foods.

[6] Granato D., Putnik P., Kovačević D.B., Santos J.S., Calado V., Rocha R.S., Cruz A.G.D., Jarvis B., Rodionova O.Y., Pomerantsev A. (2018). Trends in Chemometrics: Food Authentication, Microbiology, and Effects of Processing. Compr. Rev. Food Sci. Food Saf..

[7] Ye H., Yang J., Xiao G., Zhao Y., Li Z., Bai W., Zeng X., Dong H. (2023). A comprehensive overview of emerging techniques and chemometrics for authenticity and traceability of animal-derived food. Food Chem..

[8] Miedico O., Nardelli V., D’Amore T., Casale M., Oliveri P., Malegori C., Paglia G., Iammarino M. (2022). Identification of mechanically separated meat using multivariate analysis of 43 trace elements detected by inductively coupled mass spectrometry: A validated approach. Food Chem..

[9] Bajoub A., Bendini A., Fernández-Gutiérrez A., Carrasco-Pancorbo A. (2018). Olive oil authentication: A comparative analysis of regulatory frameworks with especial emphasis on quality and authenticity indices, and recent analytical techniques developed for their assessment. A review. Crit. Rev. Food Sci. Nutr..

[10] Zhang X.H., Gu H.W., Liu R.J., Qing X.D., Nie J.F. (2023). A comprehensive review of the current trends and recent advancements on the authenticity of honey. Food Chem X.

[11] Cichna-Markl M., Mafra I. (2023). Techniques for Food Authentication: Trends and Emerging Approaches. Foods.

[12] European Commission Knowledge Centre for Food Fraud and Quality. https://ec.europa.eu/knowledge4policy/food-fraud-quality_en.

[13] Genis D.O., Sezer B., Durna S., Boyaci I.H. (2021). Determination of milk fat authenticity in ultra-filtered white cheese by using Raman spectroscopy with multivariate data analysis. Food Chem..

[14] Białek A., Białek M., Lepionka T., Czerwonka M., Czauderna M. (2020). Chemometric Analysis of Fatty Acids Profile of Ripening Chesses. Molecules.

[15] Yazgan N.N., Genis H.E., Bulat T., Topcu A., Durna S., Yetisemiyen A., Boyaci I.H. (2020). Discrimination of milk species using Raman spectroscopy coupled with partial least squares discriminant analysis in raw and pasteurized milk. J. Sci. Food Agric..

[16] Balan B., Dhaulaniya A.S., Jamwal R., Amit, Sodhi K.K., Kelly S., Cannavan A., Singh D.K. (2020). Application of Attenuated Total Reflectance-Fourier Transform Infrared (ATR-FTIR) spectroscopy coupled with chemometrics for detection and quantification of formalin in cow milk. Vib. Spectrosc..

[17] Teixeira J.L.d.P., Caramês E.T.d.S., Baptista D.P., Gigante M.L., Pallone J.A.L. (2020). Vibrational spectroscopy and chemometrics tools for authenticity and improvement the safety control in goat milk. Food Control.

[18] Müller-Maatsch J., Alewijn M., Wijtten M., Weesepoel Y. (2021). Detecting fraudulent additions in skimmed milk powder using a portable, hyphenated, optical multi-sensor approach in combination with one-class classification. Food Control.

[19] Coimbra P.T., Bathazar C.F., Guimarães J.T., Coutinho N.M., Pimentel T.C., Neto R.P.C., Esmerino E.A., Freitas M.Q., Silva M.C., Tavares M.I.B. (2020). Detection of formaldehyde in raw milk by time domain nuclear magnetic resonance and chemometrics. Food Control.

[20] Vatavali K., Kosma I., Louppis A., Gatzias I., Badeka A.V., Kontominas M.G. (2020). Characterisation and differentiation of geographical origin of Graviera cheeses produced in Greece based on physico-chemical, chromatographic and spectroscopic analyses, in combination with chemometrics. Int. Dairy J..

[21] Da Paixao Teixeira J.L., dos Santos Carames E.T., Baptista D.P., Gigante M.L., Pallone J.A.L. (2021). Rapid adulteration detection of yogurt and cheese made from goat milk by vibrational spectroscopy and chemometric tools. J. Food Compos. Anal..

[22] Visciano P., Schirone M. (2021). Food frauds: Global incidents and misleading situations. Trends Food Sci. Technol..

[23] Macháčková K., Zelený J., Lang D., Vinš Z. (2021). Wild boar meat as a sustainable substitute for pork: A mixed methods approach. Sustainability.

[24] Siswara H.N., Erwanto Y., Suryanto E. (2022). Study of meat species adulteration in Indonesian commercial beef meatballs related to halal law implementation. Front. Sustain. Food Syst..

[25] Mortas M., Awad N., Ayvaz H. (2022). Adulteration detection technologies used for halal/kosher food products: An overview. Discov. Food.

[26] Jiang G., Sun H., Sun H., Fu Y., Li X., Wang L., Liu X. (2022). Effects of γ-aminobutyric acid on freshness and processing properties of eggs during storage. Food Res. Int..

[27] Puertas G., Cazón P., Vázquez M. (2023). A quick method for fraud detection in egg labels based on egg centrifugation plasma. Food Chem..

[28] Meng Y., Qiu N., Guyonnet V., Mine Y. (2022). Unveiling and application of the chicken egg proteome: An overview on a two-decade achievement. Food Chem..

[29] Akowuah T.O., Teye E., Hagan J., Nyandey K. (2020). Rapid and nondestructive determination of egg freshness category and marked date of lay using spectral fingerprint. J. Spectrosc..

[30] He X., Wang J., Wang Y., Wang B., Zhang J., Geng F. (2023). Quantitative lipidomic analysis of egg yolk, yolk granule, and yolk plasma. J. Food Compos. Anal..

[31] Joshi R., Lohumi S., Joshi R., Kim M.S., Qin J., Baek I., Cho B. (2020). Raman spectral analysis for non-invasive detection of external and internal parameters of fake eggs. Sens. Actuators B Chem..

[32] Gayo J., Hale S.A., Blanchard S.M. (2006). Quantitative analysis and detection of adulteration in crab meat using visible and near-infrared spectroscopy. J. Agric. Food Chem..

[33] Lv H., Xu W., You J., Xiong S. (2017). Classification of freshwater fish species by linear discriminant analysis based on near infrared reflectance spectroscopy. J. Near Infrared Spectrosc..

[34] Zhang X.Y., Hu W., Teng J., Peng H.H., Gan J.H., Wang X.C., Sun S.Q., Xu C.H., Liu Y. (2017). Rapid recognition of marine fish surimi by one-step discriminant analysis based on near-infrared diffuse reflectance spectroscopy. Int. J. Food Prop..

[35] Alamprese C., Casiraghi E. (2015). Application of FT-NIR and FT-IR spectroscopy to fish fillet authentication. LWT-Food Sci. Tech..

[36] Cozzolino D., Chree A., Scaife J.R., Murray I. (2005). Usefulness of near-infrared reflectance (NIR) spectroscopy and chemometrics to discriminate fishmeal batches made with different fish species. J. Agric. Food Chem..

[37] Sousa N., Moreira M.J., Saraiva C., De Almeida J.M. (2018). Applying fourier transform mid infrared spectroscopy to detect the adulteration of salmo salar with oncorhynchus mykiss. Foods.

[38] Chen Z., Wu T., Xiang C., Xu X., Tian X. (2019). Rapid identification of rainbow trout adulteration in Atlantic salmon by Raman spectroscopy combined with machine learning. Molecules.

[39] Rašković B., Heinke R., Rösch P., Popp J. (2016). The Potential of Raman Spectroscopy for the Classification of Fish Fillets. Food Anal. Methods.

[40] Standal I.B., Axelson D.E., Aursand M. (2010). 13C NMR as a tool for authentication of different gadoid fish species with emphasis on phospholipid profiles. Food Chem..

[41] Capuano E., Lommen A., Heenan S., de la Dura A., Rozijn M., van Ruth S. (2012). Wild salmon authenticity can be predicted by ^1^H-NMR spectroscopy. Lipid Technol..

[42] Vidal N.P., Manzanos M.J., Goicoechea E., Guillén M.D. (2012). Quality of farmed and wild sea bass lipids studied by ^1^H NMR: Usefulness of this technique for differentiation on a qualitative and a quantitative basis. Food Chem..

[43] Masoum S., Malabat C., Jalali-Heravi M., Guillou C., Rezzi S., Rutledge D.N. (2007). Application of support vector machines to ^1^H NMR data of fish oils: Methodology for the confirmation of wild and farmed salmon and their origins. Anal. Bioanal. Chem..

[44] Dalle Zotte A., Ottavian M., Concollato A., Serva L., Martelli R., Parisi G. (2014). Authentication of raw and cooked freeze-dried rainbow trout (*Oncorhynchus mykiss*) by means of near infrared spectroscopy and data fusion. Food Res. Int..

[45] He S., Sun X., Du M., Chen H., Tan M., Sun H., Zhu B. (2019). Effects of muscle protein denaturation and water distribution on the quality of false abalone (*Volutharpa ampullacea perryi*) during wet heating. J. Food Process Eng..

[46] Cropotova J., Mozuraityte R., Standal I.B., Rustad T. (2019). Assessment of lipid oxidation in Atlantic mackerel (*Scomber scombrus*) subjected to different antioxidant and sous-vide cooking treatments by conventional and fluorescence microscopy methods. Food Control.

[47] Yuan L., Dang Q., Mu J., Feng X., Gao R. (2018). Mobility and redistribution of waters within bighead carp (*Aristichthys nobilis*) heat-induced myosin gels. Int. J. Food Prop..

[48] Tavares W.P.S., Dong S., Jin W., Yang Y., Han K., Zha F., Zhao Y., Zeng M. (2018). Effect of different cooking conditions on the profiles of Maillard reaction products and nutrient composition of hairtail (*Thichiurus lepturus*) fillets. Food Res. Int..

[49] Xia K., Xu W., Huang L., Song Y., Zhu B.W., Tan M. (2018). Water dynamics of turbot flesh during frying, boiling, and stewing processes and its relationship with color and texture properties: Low-field NMR and MRI studies. J. Food Process. Preserv..

[50] Zhang H., Zhu Y., Chen S., Xu C., Yu Y., Wang X., Shi W. (2018). Determination of the effects of different high-temperature treatments on texture and aroma characteristics in Alaska pollock surimi. Food Sci. Nutr..

[51] Ovissipour M., Shiroodi S.G., Rasco B., Tang J., Sablani S.S. (2018). Electrolyzed water and mild-thermal processing of Atlantic salmon (*Salmo salar*): Reduction of Listeria monocytogenes and changes in protein structure. Int. J. Food Microbiol..

[52] Hu L., Ren S., Shen Q., Ye X., Chen J., Ling J. (2018). Protein oxidation and proteolysis during roasting and in vitro digestion of fish (*Acipenser gueldenstaedtii*). J. Sci. Food Agric..

[53] Wold J.P. (2016). On-line and non-destructive measurement of core temperature in heat treated fish cakes by NIR hyperspectral imaging. Innov. Food Sci. Emerg. Technol..

[54] Ovissipour M., Rasco B., Tang J., Sablani S. (2017). Kinetics of protein degradation and physical changes in thermally processed Atlantic salmon (*Salmo salar*). Food Βioprocess Τechnol..

[55] Belkhanchi H., Ziat Y., Hammi M., Ifguis O. (2023). Formulation, optimization of a poultry feed and analysis of spectrometry, biochemical composition and energy facts. S. Afr. J. Chem. Eng..

[56] El-Tahawy A.A.S., Taha A.E., Adel S.A. (2017). Effect of flock size on the productive and economic efficiency of Ross 308 and Cobb 500 broilers. Eur. Poult. Sci..

[57] Gado A., Ellakany H., Elbestawy A., Abd El-Hack M., Khafaga A., Taha A., Arif M., Mahgoub S. (2019). Herbal medicine additives as powerful agents to control and prevent avian influenza virus in poultry—A review. Ann. Anim. Sci..

[58] OECD-FAO (2021). OECD-FAO Agricultural Outlook 2021–2030.

[59] Altmann B.A., Rosenau S. (2022). Spirulina as animal feed: Opportunities and challenges. Foods.

[60] Khan R.U., Fatima A., Naz S., Ragni M., Tarricone S., Tufarelli V. (2022). Perspective, opportunities and challenges in using fennel (*Foeniculum vulgare*) in poultry health and production as an eco-friendly alternative to antibiotics: A review. Antibiotics.

[61] Bryant R.B., Endale D.M., Spiegal S.A., Flynn K.C., Meinen R.J., Cavigelli M.A., Kleinman P.J. (2022). Poultry manureshed management: Opportunities and challenges for a vertically integrated industry. J. Environ. Qual..

[62] Abdelli N., Sola Oriol D., Perez J.F. (2021). Phytogenic feed additives in poultry: Achievements, prospective and challenges. Animals.

[63] Kpomasse C.C., Oke O.E., Houndonougbo F.M., Tona K. (2021). Broiler production challenges in the tropics: A review. Vet. Med. Sci..

[64] Alhotan R.A. (2021). Commercial poultry feed formulation: Current status, challenges, and future expectations. Worlds Poult. Sci..

[65] Aboah J., Enahoro D. (2022). A systems thinking approach to understand the drivers of change in backyard poultry farming system. Agric. Syst..

[66] Diaz-Sanchez S., Moscoso S., Solis de los Santos F., Andino A., Hanning I. (2015). Antibiotic use in poultry: A driving force for organic poultry production. Food Protction Trends.

[67] Erinle T.J., Adewole D.I. (2022). Fruit pomaces—Their nutrient and bioactive components, effects on growth and health of poultry species, and possible optimization techniques. Anim. Nutr..

[68] Madkour M., Salman F.M., El-Wardany I., Abdel-Fattah S.A., Alagawany M., Hashem N.M., Dhama K. (2022). Mitigating the detrimental effects of heat stress in poultry through thermal conditioning and nutritional manipulation. J. Therm. Biol..

[69] Gürbüz M., Korkmaz B.İ.O. (2022). The anti-campylobacter activity of eugenol and its potential for poultry meat safety: A review. Food Chem..

[70] Havenstein G.B., Crittenden L.B., Petitte J.N., Qureshi H.A., Foster D.N. (1992). Application of biotechnology in the poultry industry. Anim. Biotechnol..

[71] Havenstein G.B., Ferket P.R., Qureshi M.A. (2003). Carcass composition and yield of 1957 versus 2001 broilers when fed representative 1957 and 2001 broiler diets. Poulry Sci..

[72] Bolder N.M. (2007). Microbial challenges of poultry meat production. World’s Poult. Sci. J..

[73] Vandeplas S., Dauphin R.D., Beckers Y., Thonart P., Thewis A. (2010). Salmonella in chicken: Current and developing strategies to reduce contamination at farm level. J. Food Prot..

[74] Cox N.A., Cason J.A., Richardson L.J. (2011). Minimization of Salmonella contamination on raw poultry. Annu. Rev. Food Sci. Technol..

[75] Chao K., Kim M.S., Chan D.E. (2014). Control interface and tracking control system for automated poultry inspection. Comput. Stand. Interfaces.

[76] Ricke S.C. (2017). Insights and challenges of Salmonella infection of laying hens. Curr. Opin. Food Sci..

[77] Ponka R., Goudoum A., Tchungouelieu A.C., Fokou E. (2016). Evaluation nutritionnelle de quelques ingrédients entrant dans la formulation alimentaire des poules pondeuses et porcs d’une ferme d’élevage au Nord-Ouest Cameroun. Int. J. Biol. Chem. Sci..

[78] Biesek J., Banaszak M., Wlaźlak S., Adamski M. (2022). The effect of partial replacement of milled finisher feed with wheat grains on the production efficiency and meat quality in broiler chickens. Poult. Sci..

[79] Santos R.R., Velkers F.C., Vernooij J.C.M., Star L., Heerkens J.L.T., van Harn J., de Jong I.C. (2022). Nutritional interventions to support broiler chickens during Eimeria infection. Poul. Sci..

[80] Nasir N.A.N.M., Kamaruddin S.A., Zakarya I.A., Islam A.K.M.A. (2022). Sustainable alternative animal feeds: Recent advances and future perspective of using azolla as animal feed in livestock, poultry and fish nutrition. Sustain. Chem. Pharm..

[81] Kogut M.H. (2022). Role of diet-microbiota interactions in precision nutrition of the chicken: Facts, gaps, and new concepts. Poul. Sci..

[82] Peng Z., Yan L., Wei L., Gao X., Shi L., Ren T., Han Y. (2022). Effect of dietary chicken gut meal levels on growth performance, plasma biochemical parameters, digestive ability and fillet quality of *Cyprinus carpio*. Aquac. Rep..

[83] Kleter G., McFarland S., Bach A., Bernabucci U., Bikker P., Busani L., Einspanier R. (2018). Surveying selected European feed and livestock production chains for features enabling the case-specific post-market monitoring of livestock for intake and potential health impacts of animal feeds derived from genetically modified crops. Food Chem. Toxicol..

[84] EFSA (2011). Guidance for risk assessment of food and feed from genetically modified plants. EFSA J..

[85] EU Commission Implementing Regulation (EU) No 503/2013 of 3 April 2013 on Applications for Authorisation of Genetically Modified Food and Feed in Accordance with Regulation (EC) No 1829/2003 of the European Parliament and of the Council and Amending Commission Regulations (EC) No 641/2004 and (EC) No 1981/2006. Off. J. Eur. Union L157, 2013. pp. 1–48. http://eur-lex.europa.eu/legal-content/EN/ALL/?uri=CELEX%3A32013R0503.

[86] Chen H., Tan C., Lin Z. (2019). Non-destructive identification of native egg by near-infrared spectroscopy and data driven-based class-modeling. Spectrochim. Acta Part. A Mol. Biomol. Spectrosc..

[87] Wu D., He Y., Feng S. (2008). Short-wave near-infrared spectroscopy analysis of major compounds in milk powder and wavelength assignment. Anal. Chim. Acta.

[88] Cozzolino D., Espiñeira M., Santaclara F.J. (2016). Near infrared spectroscopy and food authenticity. Advances in Food Traceability Techniques and Technologies.

[89] Domingo E., Tirelli A.A., Nunes C.A., Guerreiro M.C., Pinto S.M. (2014). Melamine detection in milk using vibrational spectroscopy and chemometrics analysis: A review. Food Res. Int..

[90] Chen H., Lin Z., Wu H., Wang L., Wu T., Tan C. (2015). Diagnosis of colorectal cancer by near-infrared optical fiber spectroscopy and random forest. Spectrochim. Acta Part. A Mol. Biomol. Spectrosc..

[91] Lyndgaard C.B., Engelsen S.B., van den Berg F.W. (2012). Real-time modeling of milk coagulation using in-line near infrared spectroscopy. J. Food Eng..

[92] Chen H., Tan C., Lin Z., Wu T. (2017). Detection of melamine adulteration in milk by near-infrared spectroscopy and one-class partial least squares. Spectrochim. Acta Part A Mol. Biomol. Spectrosc..

[93] Rogers K.M., Van Ruth S., Alewijn M., Philips A., Rogers P. (2015). Verification of egg farming systems from the Netherlands and New Zealand using stable isotopes. J. Agric. Food Chem..

[94] Bandoniene D., Walkner C., Zettl D., Meisel T. (2018). Rare earth element labeling as a tool for assuring the origin of eggs and poultry products. J. Agric. Food Chem..

[95] Li W., Huang W., Fan D., Gao X., Zhang X., Meng Y., Liu T.C. (2023). Rapid quantification of goat milk adulteration with cow milk using Raman spectroscopy and chemometrics. Anal. Methods.

[96] Huang W., Fan D., Li W., Meng Y., Liu T.C. (2022). Rapid evaluation of milk acidity and identification of milk adulteration by Raman spectroscopy combined with chemometrics analysis. Vib. Spectrosc..

[97] Pellegrino L., Hogenboom J.A., Rosi V., D’Incecco P. (2021). Evaluating the Authenticity of the Raw-Milk Cheese Fontina (PDO) with Respect to Similar Cheeses. Foods.

[98] Tarapoulouzi M., Theocharis C.R. (2022). Discrimination of Cheddar, Kefalotyri, and Halloumi cheese samples by the chemometric analysis of Fourier transform infrared spectroscopy and proton nuclear magnetic resonance spectra. J. Food Process. Eng..

[99] Tarapoulouzi M., Theocharis C.R. (2023). Discrimination of Anari Cheese Samples in Comparison with Halloumi Cheese Samples Regarding the Origin of the Species by FTIR Measurements and Chemometrics. Analytica.

[100] Strani L., Grassi S., Alamprese C., Casiraghi E., Ghiglietti R., Locci F., Pricca N., De Juan A. (2021). Effect of physicochemical factors and use of milk powder on milk rennet-coagulation: Process understanding by near infrared spectroscopy and chemometrics. Food Control.

[101] Du L., Lu W., Zhang Y., Gao B., Yu L. (2020). Detection of milk powder in liquid whole milk using hydrolyzed peptide and intact protein mass spectral fingerprints coupled with data fusion technologies. Food Sci. Nutr..

[102] Potočnik D., Nečemer M., Perišić I., Jagodic M., Mazej D., Camin F., Eftimov T., Strojnik L., Ogrinc N. (2020). Geographical verification of Slovenian milk using stable isotope ratio, multi-element and multivariate modelling approaches. Food Chem..

[103] Hamzić Gregorčič S., Potočnik D., Camin F., Ogrinc N. (2020). Milk Authentication: Stable Isotope Composition of Hydrogen and Oxygen in Milks and Their Constituents. Molecules.

[104] O’Sullivan R., Monahan F.J., Bahar B., Kirwan L., Pierce K., O’Shea A., McElroy S., Malone F., Hanafin B., Molloy S. (2021). Stable isotope profile (C, N, O, S) of Irish raw milk: Baseline data for authentication. Food Control.

[105] Chung I., Kim J., Yang Y., An Y., Kim S., Kwon C., Kim S. (2020). A case study for geographical indication of organic milk in Korea using stable isotope ratios-based chemometric analysis. Food Control.

[106] Tarapoulouzi M., Kokkinofta R., Theocharis C.R. (2020). Chemometric analysis combined with FTIR spectroscopy of milk and Halloumi cheese samples according to species’ origin. Food Sci. Nutr..

[107] Nikolaou P., Deskoulidis E., Topoglidis E., Kakoulidou A.T., Tsopelas F. (2020). Application of chemometrics for detection and modeling of adulteration of fresh cow milk with reconstituted skim milk powder using voltammetric fingerpriting on a graphite/SiO_2_ hybrid electrode. Talanta.

[108] Bergamaschi M., Cipolat-Gotet C., Cecchinato A., Schiavon S., Bittante G. (2020). Chemometric authentication of farming systems of origin of food (milk and ripened cheese) using infrared spectra, fatty acid profiles, flavor fingerprints, and sensory descriptions. Food Chem..

[109] Visoka Y., Majadi M., Kovacs Z., Gecaj R.M. (2023). Utilizing NearInfrared Spectroscopy for Discriminant Analysis of Goat Milk Composition across Diverse Breeds and Lactation Seasons. Biol. Life Sci. Forum.

[110] Li Vigni M., Durante C., Michelini S., Nocetti M., Cocchi M. (2020). Preliminary Assessment of Parmigiano Reggiano Authenticity by Handheld Raman Spectroscopy. Foods.

[111] Di Donato F., Biancolillo A., Mazzulli D., Rossi L., D’Archivio A.A. (2021). HS-SPME/GC–MS volatile fraction determination and chemometrics for the discrimination of typical Italian Pecorino cheeses. Microchem. J..

[112] Tarapoulouzi M., Theocharis C.R. (2019). Discrimination of Cheddar and Kefalotyri Cheese Samples: Analysis by Chemometrics of Proton-NMR and FTIR Spectra. J. Agric. Sci. Technol..

[113] Hanganu A., Chira N. (2021). When detection of dairy food fraud fails: An alternate approach through proton nuclear magnetic resonance spectroscopy. J. Dairy Sci..

[114] Temizkan R., Can A., Dogan M.A., Mortas M., Ayvaz H. (2020). Rapid detection of milk fat adulteration in yoghurts using near and mid-infrared spectroscopy. Int. Dairy J..

[115] Limm W., Karunathilaka S.R., Yakes B.J., Mossoba M.M. (2018). A portable mid-infrared spectrometer and a non-targeted chemometric approach for the rapid screening of economically motivated adulteration of milk powder. Int. Dairy J..

[116] Tarapoulouzi M., Theocharis C. (2019). Application of Chemometrics to the Combined Data from 1H-NMR and FTIR Spectra: Discrimination of Cheddar and Kefalotyri Cheese Samples.

[117] Tolentino I.C.D.S.A., de Jesus J.C., Conceição D.G., Onelli R.R.V., Reis L.C.C., Santos L.S., Ferrão S.P.B. (2023). Use of MIR spectroscopy associated with chemometric techniques to verify the authenticity of prato cheese. Int. Dairy J..

[118] Khan M., Khan M.I., Sahar A., Jamil A. (2020). Predicting authenticity and physicochemical characteristics of meat through FT-IR spectroscopy coupled with multivariate analysis. Pak. J. Agric. Sci..

[119] Varrà M.O., Fasolato L., Serva L., Ghidini S., Novelli E., Zanardi E. (2020). Use of near infrared spectroscopy coupled with chemometrics for fast detection of irradiated dry fermented sausages. Food Control.

[120] Achata E.M., Oliveira M., Esquerre C.A., Tiwari B.K., O’Donnell C.P. (2020). Visible and NIR hyperspectral imaging and chemometrics for prediction of microbial quality of beef Longissimus dorsi muscle under simulated normal and abuse storage conditions. LWT.

[121] Rebellato A.P., Caramês E.T.d.S., Moraes P.P.d., Pallone J.A.L. (2020). Minerals assessment and sodium control in hamburger by fast and green method and chemometric tools. LWT.

[122] Leng T., Li F., Xiong L., Xiong Q., Zhu M., Chen Y. (2020). Quantitative detection of binary and ternary adulteration of minced beef meat with pork and duck meat by NIR combined with chemometrics. Food Control.

[123] Cozzolino D., Bureš D., Hoffman L.C. (2023). Evaluating the Use of a Similarity Index (SI) Combined with near Infrared (NIR) Spectroscopy as Method in Meat Species Authenticity. Foods.

[124] Hoffman L., Ingle P., Khole A.H., Zhang S., Yang Z., Beya M., Bureš D., Cozzolino D. (2023). Discrimination of lamb (*Ovis aries*), emu (*Dromaius novaehollandiae*), camel (*Camelus dromedarius*) and beef (*Bos taurus*) binary mixtures using a portable near infrared instrument combined with chemometrics. Spectrochim. Acta Part A Mol. Biomol. Spectrosc..

[125] Parastar H., van Kollenburg G., Weesepoel Y., van den Doel A., Buydens L., Jansen J. (2020). Integration of handheld NIR and machine learning to “Measure & Monitor” chicken meat authenticity. Food Control.

[126] Kalinichenko A., Arseniyeva L. (2020). Electronic nose combined with chemometric approaches to assess authenticity and adulteration of sausages by soy protein. Sens. Actuators B Chem..

[127] Ahda M., Guntarti A., Kusbandari A., Melianto Y. (2021). Authenticity Analysis of Beef Meatball Adulteration with Wild Boar using FTIR Spectroscopy Combined with Chemometrics. J. Microbiol. Biotechnol. Food Sci..

[128] Robert C., Fraser-Miller S.J., Jessep W.T., Bain W.E., Hicks T.M., Ward J.F., Craigie C.R., Loeffen M., Gordon K.C. (2021). Rapid discrimination of intact beef, venison and lamb meat using Raman spectroscopy. Food Chem..

[129] Keshavarzi Z., Banadkoki S.B., Faizi M., Zolghadri Y., Shirazi F.H. (2020). Comparison of transmission FTIR and ATR spectra for discrimination between beef and chicken meat and quantification of chicken in beef meat mixture using ATR-FTIR combined with chemometrics. J. Food Sci. Technol..

[130] Ghazanfari M., Motalebi A., Hosseini H., Rokni N. (2021). Use of Fourier transform infrared spectroscopy (FTIR) and PCA, PLS-DA and SIMCA techniques for the authenticity of hamburger meats. Veterinary Research Forum.

[131] Song X., Canellas E., Nerin C. (2021). Screening of volatile decay markers of minced pork by headspace-solid phase microextraction–gas chromatography–mass spectrometry and chemometrics. Food Chem..

[132] Nunes K.M., Andrade M.V.O., Almeida M.R., Sena M.M. (2020). A soft discriminant model based on mid-infrared spectra of bovine meat purges to detect economic motivated adulteration by the addition of non-meat ingredients. Food Anal. Methods.

[133] Windarsih A., Bakar N.K.A., Dachriyanus, Yuliana N.D., Riswanto F.D.O., Rohman A. (2023). Analysis of Pork in Beef Sausages Using LC-Orbitrap HRMS Untargeted Metabolomics Combined with Chemometrics for Halal Authentication Study. Molecules.

[134] Ahda M., Guntarti A., Kusbandari A., Andoyo Nugroho H. (2023). Identification of Adulterated Sausage Products by Pork using FTIR and GC-MS Combined with Chemometrics. J. Chem. Health Risks.

[135] Totaro M.P., Squeo G., De Angelis D., Pasqualone A., Caponio F., Summo C. (2023). Application of NIR spectroscopy coupled with DD-SIMCA class modelling for the authentication of pork meat. J. Food Compos. Anal..

[136] An J.M., Hur S.H., Kim H., Lee J.H., Kim Y.K., Sim K.S., Lee S.E., Kim H.J. (2023). Determination of the geographical origin of chicken (breast and drumstick) using ICP-OES and ICP-MS: Chemometric analysis. Food Chem..

[137] Cermakova E., Lencova S., Mukherjee S., Horka P., Vobruba S., Demnerova K., Zdenkova K. (2023). Identification of Fish Species and Targeted Genetic Modifications Based on DNA Analysis: State of the Art. Foods.

[138] Kotsanopoulos K.V., Exadactylos A., Gkafas G.A., Martsikalis P.V., Parlapani F.F., Boziaris I.S., Arvanitoyannis I.S. (2021). The use of molecular markers in the verification of fish and seafood authenticity and the detection of adulteration. Compr. Rev. Food Sci. Food Saf..

[139] Lin T.C., Hsiao W.V., Han S.J., Joung S.J., Shiao J.C. (2021). A direct multiplex loop-mediated isothermal amplification method to detect three CITES-listed shark species. Aquat. Conserv. Mar. Freshw..

[140] But G.W.C., Wu H.Y., Shao K.T., Shaw P.C. (2020). Rapid detection of CITES-listed shark fin species by loop-mediated isothermal amplification assay with potential for field use. Sci. Rep..

[141] Randhawa S.S., Pawar R. (2021). Fish genomes: Sequencing trends, taxonomy and influence of taxonomy on genome attributes. J. Appl. Ichthyol..

[142] Guan F., Jin Y.T., Zhao J., Xu A.C., Luo Y.Y. (2018). A PCR method that can be further developed into PCR-RFLP assay for eight animal species identification. J. Anal. Methods Chem..

[143] Teletchea F. (2009). Molecular identification methods of fish species: Reassessment and possible applications. Rev. Fish. Biol. Fis..

[144] Ballin N.Z., Vogensen F.K., Karlsson A.H. (2009). Species determination–Can we detect and quantify meat adulteration?. Meat Sci..

[145] Yao L., Qu M., Jiang Y., Guo Y., Li N., Li F., Tan Z., Wang L. (2022). The development of genus-specific and species-specific real-time PCR assays for the authentication of Patagonian toothfish and Antarctic toothfish in commercial seafood products. J. Sci. Food Agric..

[146] Afzaal M., Saeed F., Hussain M., Shahid F., Siddeeg A., Al-Farga A. (2022). Proteomics as a promising biomarker in food authentication, quality and safety: A review. Food Sci. Nutr..

[147] Tozzo P., Mazzobel E., Marcante B., Delicati A., Caenazzo L. (2022). Touch DNA sampling methods: Efficacy evaluation and systematic review. Int. J. Mol. Sci..

[148] Gall-Duncan T., Sato N., Yuen R.K., Pearson C.E. (2022). Advancing genomic technologies and clinical awareness accelerates discovery of disease-associated tandem repeat sequences. Genome Res..

[149] Zhang J., Cai Z. (2007). The application of DGGE and AFLP-derived SCAR for discrimination between Atlantic salmon (*Salmo salar*) and rainbow trout (*Oncorhynchus mykiss*). Food Control.

[150] Maldini M., Marzano F.N., Fortes G.G., Papa R., Gandolfi G. (2006). Fish and seafood traceability based on AFLP markers: Elaboration of a species database. Aquaculture.

[151] Akasaki T., Yanagimoto T., Yamakami K., Tomonaga H., Sato S. (2006). Species identification and PCR-RFLP analysis of cytochrome b gene in cod fish (order Gadiformes) products. J. Food Sci..

[152] Espineira M., Gonzalez-Lavin N., Vieites J.M., Santaclara F.J. (2008). Authentication of anglerfish species (*Lophius* spp.) by means of polymerase chain reaction—Restriction fragment length polymorphism (PCR−RFLP) and forensically informative nucleotide sequencing (FINS) methodologies. J. Agric. Food Chem..

[153] Yu Z., Guo X. (2004). Genetic analysis of selected strains of eastern oyster (*Crassostrea virginica* Gmelin) using AFLP and microsatellite markers. Mar. Biotechnol..

[154] Lin W.F., Hwang D.F. (2007). Application of PCR-RFLP analysis on species identification of canned tuna. Food Control.

[155] Yao L., Lu J., Qu M., Jiang Y., Li F., Guo Y., Wang L., Zhai Y. (2020). Methodology and application of PCR-RFLP for species identification in tuna sashimi. Food Sci. Nutr..

[156] Hwang C.C., Lin C.M., Huang C.Y., Huang Y.L., Kang F.C., Hwang D.F., Tsai Y.H. (2012). Chemical characterisation, biogenic amines contents, and identification of fish species in cod and escolar steaks, and salted escolar roe products. Food Control.

[157] Li M., Zhang K.Y.B., But P.P.H., Shaw P.C. (2011). Forensically informative nucleotide sequencing (FINS) for the authentication of Chinese medicinal materials. Chin. Med..

[158] Santaclara F.J., Espiñeira M., Vieites J.M. (2007). Genetic identification of squids (families Ommastrephidae and Loliginidae) by PCR–RFLP and FINS methodologies. J. Agric. Food Chem..

[159] Mukherjee S., Hanak P., Jilkova D., Musilova Z., Horka P., Lerch Z., Zdenkova K., Cermakova E. (2023). Simultaneous detection and quantification of two European anglerfishes by novel genomic primer. J. Food Compos. Anal..

[160] Li Q., Xue H., Fei Y., Cao M., Xiong X., Xiong X., Yang Y., Wang L. (2022). Visual detection of rainbow trout (*Oncorhynchus mykiss*) and Atlantic salmon (*Salmo salar*) simultaneously by duplex loop-mediated isothermal amplification. Food Chem. Mol. Sci..

[161] Xu W., Fu M., Huang M., Cui X., Li Y., Cao M., Wang L., Xiong X., Xiong X. (2021). Duplex real-time PCR combined with melting curve analysis for rapid detection of Atlantic salmon (*Salmo salar*) and rainbow trout (*Oncorhynchus mykiss*). J. Food Compos. Anal..

[162] Xiong X., Huang M., Xu W., Li Y., Cao M., Xiong X. (2021). Using real time fluorescence loop-mediated isothermal amplification for rapid species authentication of Atlantic salmon (*Salmo salar*). J. Food Compos. Anal..

[163] Xiong X., Yuan F., Huang M., Xiong X. (2020). Exploring the possible reasons for fish fraud in China based on results from monitoring sardine products sold on Chinese markets using DNA barcoding and real time PCR. Food Addit. Contam. Part A.

[164] Xiong X., Huang M., Xu W., Cao M., Li Y., Xiong X. (2020). Tracing Atlantic Salmon (*Salmo salar*) in processed fish products using the novel loop-mediated isothermal amplification (LAMP) and PCR assays. Food Anal. Methods.

[165] Xiong X., Huang M., Xu W., Li Y., Cao M., Xiong X. (2020). Rainbow trout (*Oncorhynchus mykiss*) identification in processed fish products using loop-mediated isothermal amplification and polymerase chain reaction assays. J. Sci. Food Agric..

[166] Shi R., Xiong X., Huang M., Xu W., Li Y., Cao M., Xiong X. (2020). High resolution melting (HRM) analysis of a 12S rRNA mini barcode as a novel approach for codfish species authentication in processed fish products. Eur. Food Res. Technol..

[167] Soga K., Nakamura K., Ishigaki T., Kimata S., Ohmori K., Kishine M., Mano J., Takabatake R., Kitta K., Nagoya H. (2019). Data representing applicability of developed growth hormone 1 (GH1) gene detection method for detecting Atlantic salmon (*Salmo salar*) at high specificity to processed salmon commodities. Data Br..

[168] Fernandes T.J., Costa J., Oliveira M.B.P., Mafra I. (2018). Exploiting 16S rRNA gene for the detection and quantification of fish as a potential allergenic food: A comparison of two real-time PCR approaches. Food Chem..

[169] Holman L.E., Onoufriou A., Hillestad B., Johnston I.A. (2017). A workflow used to design low density SNP panels for parentage assignment and traceability in aquaculture species and its validation in Atlantic salmon. Aquaculture.

[170] Leonardo R., Nunes R.S.C., Maria L., Conte-Junior C.A., Del Aguila E.M., Paschoalin V.M. (2016). Molecular testing on sardines and rulings on the authenticity and nutritional value of marketed fishes: An experience report in the state of Rio de Janeiro, Brazil. Food Control.

[171] Madanayake N.H., Hossain A., Adassooriya N.M. (2021). Nanobiotechnology for agricultural sustainability, and food and environmental safety. Qual. Assur. Saf. Crops Foods.

[172] Tang X., Liu Y., Hou H., You T. (2011). A nonenzymatic sensor for xanthine based on electrospun carbon nanofibers modified electrode. Talanta.

[173] Heising J.K., Bartels P.V., Van Boekel M.A.J.S., Dekker M. (2014). Non-destructive sensing of the freshness of packed cod fish using conductivity and pH electrodes. J. Food Eng..

[174] Chang L.Y., Chuang M.Y., Zan H.W., Meng H.F., Lu C.J., Yeh P.H., Chen J.N. (2017). One-minute fish freshness evaluation by testing the volatile amine gas with an ultrasensitive porous-electrode-capped organic gas sensor system. ACS Sens..

[175] Li C., Hao J., Wu K. (2019). Triethylamine-controlled Cu-BTC frameworks for electrochemical sensing fish freshness. Anal. Chim. Acta.

[176] Thandavan K., Gandhi S., Sethuraman S., Rayappan J.B.B., Krishnan U.M. (2013). Development of electrochemical biosensor with nano-interface for xanthine sensing–A novel approach for fish freshness estimation. Food Chem..

[177] Narang J., Malhotra N., Singhal C., Pundir C.S. (2017). Evaluation of freshness of fishes using MWCNT/TiO_2_ nanobiocomposites based biosensor. Food Anal. Methods.

[178] Borisova B., Sánchez A., Jiménez-Falcao S., Martín M., Salazar P., Parrado C., Illalonga R. (2016). Reduced graphene oxide-carboxymethylcellulose layered with platinum nanoparticles/PAMAM dendrimer/magnetic nanoparticles hybrids. Application to the preparation of enzyme electrochemical biosensors. Sens. Actuators B Chem..

[179] Apetrei I.M., Apetrei C. (2016). Amperometric biosensor based on diamine oxidase/platinum nanoparticles/graphene/chitosan modified screen-printed carbon electrode for histamine detection. Sensors.

[180] Torre R., Costa-Rama E., Nouws H.P., Delerue-Matos C. (2020). Diamine oxidase-modified screen-printed electrode for the redox-mediated determination of histamine. J. Anal. Sci. Technol..

[181] Nguyen T.V., Van Chuyen H. (2021). Prevalence, determination, and control of histamine formation in food concerning food safety aspect. Qual. Assur. Saf. Crops Foods.

[182] Pierini G.D., Robledo S.N., Zon M.A., Di Nezio M.S., Granero A.M., Fernandez H. (2018). Development of an electroanalytical method to control quality in fish samples based on an edge plane pyrolytic graphite electrode. Simultaneous determination of hypoxanthine, xanthine and uric acid. Microchem. J..

[183] Tan J., Li M.F. (2021). Rapid and nondestructive identification of Belgian and Netherlandish Trappist beers by front-face synchronous fluorescence spectroscopy coupled with multiple statistical analysis. Qual. Assur. Saf. Crops Foods.

[184] Guohua H., Lvye W., Yanhong M., Lingxia Z. (2012). Study of grass carp (*Ctenopharyngodon idellus*) quality predictive model based on electronic nose. Sens. Actuators B Chem..

[185] Semeano A.T., Maffei D.F., Palma S., Li R.W., Franco B.D., Roque A.C., Gruber J. (2018). Tilapia fish microbial spoilage monitored by a single optical gas sensor. Food Control.

[186] Haugen J.E., Chanie E., Westad F., Jonsdottir R., Bazzo S., Labreche S., Olafsdottir G. (2006). Rapid control of smoked Atlantic salmon (*Salmo salar*) quality by electronic nose: Correlation with classical evaluation methods. Sens. Actuators B Chem..

[187] El Barbri N., Mirhisse J., Ionescu R., El Bari N., Correig X., Bouchikhi B., Llobet E. (2009). An electronic nose system based on a micro-machined gas sensor array to assess the freshness of sardines. Sens. Actuators B Chem..

[188] Grassi S., Benedetti S., Opizzio M., di Nardo E., Buratti S. (2019). Meat and fish freshness assessment by a portable and simplified electronic nose system (Mastersense). Sensors.

[189] Gil L., Barat J.M., Escriche I., Garcia-Breijo E., Martínez-Máñez R., Soto J. (2008). An electronic tongue for fish freshness analysis using a thick-film array of electrodes. Microchim. Acta.

[190] Barat J.M., Gil L., García-Breijo E., Aristoy M.C., Toldrá F., Martínez-Máñez R., Soto J. (2008). Freshness monitoring of sea bream (Sparus aurata) with a potentiometric sensor. Food Chem..

[191] Miao H., Liu Q., Bao H., Wang X., Miao S. (2017). Effects of different freshness on the quality of cooked tuna steak. Innov. Food Sci. Emerg. Technol..

[192] Pattarapon P., Zhang M., Bhandari B., Gao Z. (2018). Effect of vacuum storage on the freshness of grass carp (*Ctenopharyngodon idella*) fillet based on normal and electronic sensory measurement. J. Food Process. Preserv..

[193] Kiran E., Kaur K., Aggarwal P. (2022). Artificial senses and their fusion as a booming technique in food quality assessment—A review. Qual. Assur. Saf. Crops Foods.

[194] Sun D.W. (2016). Computer Vision Technology for Food Quality Evaluation.

[195] Shi C., Qian J., Han S., Fan B., Yang X., Wu X. (2018). Developing a machine vision system for simultaneous prediction of freshness indicators based on tilapia (*Oreochromis niloticus*) pupil and gill color during storage at 4 C. Food Chem..

[196] Balaban M.Ö., Alçiçek Z. (2015). Use of polarized light in image analysis: Application to the analysis of fish eye color during storage. LWT-Food Sci. Technol..

[197] Issac A., Dutta M.K., Sarkar B. (2017). Computer vision based method for quality and freshness check for fish from segmented gills. Comput. Electron. Agric..

